# Critical review of the use and scientific basis of forensic gait analysis

**DOI:** 10.1080/20961790.2018.1503579

**Published:** 2018-10-09

**Authors:** Nina M. van Mastrigt, Kevin Celie, Arjan L. Mieremet, Arnout C. C. Ruifrok, Zeno Geradts

**Affiliations:** a Department of Digital Technology and Biometry, Netherlands Forensic Institute, The Hague, The Netherlands;; b Intelligent Information Systems, University of Amsterdam, Amsterdam, The Netherlands

**Keywords:** Forensic science, forensic gait analysis, validation, biometric characteristics, image analysis, video analysis, survey, gait recognition

## Abstract

This review summarizes the scientific basis of forensic gait analysis and evaluates its use in the Netherlands, United Kingdom and Denmark, following recent critique on the admission of gait evidence in Canada. A useful forensic feature is (1) measurable, (2) consistent within and (3) different between individuals. Reviewing the academic literature, this article found that (1) forensic gait features can be quantified or observed from surveillance video, but research into accuracy, validity and reliability of these methods is needed; (2) gait is variable within individuals under differing and constant circumstances, with speed having major influence; (3) the discriminative strength of gait features needs more research, although clearly variation exists between individuals. Nevertheless, forensic gait analysis has contributed to several criminal trials in Europe in the past 15 years. The admission of gait evidence differs between courts. The methods are mainly observer-based: multiple gait analysts (independently) assess gait features on video footage of a perpetrator and suspect. Using gait feature databases, likelihood ratios of the hypotheses that the observed individuals have the same or another identity can be calculated. Automated gait recognition algorithms calculate a difference measure between video clips, which is compared with a threshold value derived from a video gait recognition database to indicate likelihood. However, only partly automated algorithms have been used in practice. We argue that the scientific basis of forensic gait analysis is limited. However, gait feature databases enable its use in court for supportive evidence with relatively low evidential value. The recommendations made in this review are (1) to expand knowledge on inter- and intra-subject gait variabilities, discriminative strength and interdependency of gait features, method accuracies, gait feature databases and likelihood ratio estimations; (2) to compare automated and observer-based gait recognition methods; to design (3) an international standard method with known validity, reliability and proficiency tests for analysts; (4) an international standard gait feature data collection method resulting in database(s); (5) (inter)national guidelines for the admission of gait evidence in court; and (6) to decrease the risk for cognitive and contextual bias in forensic gait analysis. This is expected to improve admission of gait evidence in court and judgment of its evidential value. Several ongoing research projects focus on parts of these recommendations.

## Introduction

No doubt exists about differences in human gait: most people remember instances in which they recognized friends or relatives by their walk. Gait is defined as the pattern of movement utilized during locomotion [1]. It is a cyclic activity which is easily captured on video, even from a distance. Since the amount of surveillance cameras in public environment has grown, the chance of retrieving video footage of walking perpetrators or suspects has increased. Forensic gait analysis is mostly considered if video footage contains no strong biometric clues for identification. The presence, absence or size of features derived from the gait of a perpetrator and suspect(s) can then serve as evidence. However, forensic gait analysis methods are not (yet) capable of identification. Therefore, gait is only used as supportive evidence.

Forensic gait analysis has been used as supportive evidence in criminal cases in the United Kingdom for more than 15 years [2–5] and in Denmark for more than 10 years [6]. In the Netherlands, gait analysis has been performed rarely in the past 20 years. However, two recent criminal cases renewed interest in the topic in the Netherlands.

In the academic literature, different approaches have been proposed for analysing gait in a forensic context. The computer vision approach is to design algorithms for automated gait recognition from video footage [3,4,7–9]. Requiring no or limited user intervention, the algorithm calculates gait features and compares them between perpetrator and suspect(s). In observer-based methods [6,10–12], gait analysts systematically score the presence or absence of certain gait features and compare these between perpetrator and suspect(s). The latter approach has been used in several criminal cases [6,10,13–15].

The admission of gait analysis as evidence has recently been criticized in Canada [16]. Main concerns of Edmond and Cunliffe [16,17] are the validity, reliability and scientific basis of forensic gait analysis and the inability of courts to judge the expertise of expert witnesses and the evidential value of their conclusions. Although the conclusions of Edmond and Cunliffe are based on only two cases, their concerns reinforce the need for a review of the scientific basis of forensic gait analysis and an evaluation of the use of forensic gait analysis in practice as reported in scientific literature and using a survey.

In this review, we investigate the scientific basis and use of forensic gait analysis. The first part reviews the scientific basis of forensic gait analysis: what is known about intra- and inter-variabilities of gait and which forensic gait analysis methods have been proposed? In the second part of this review, we present the results of a survey to forensic gait analysis in practice. Finally, recommendations for research into and appropriate use of forensic gait analysis will be made.

## Part I: the scientific basis of forensic gait analysis

In forensic gait analysis, comparisons are made between gait features of a perpetrator and suspect(s). For a feature to be useful in differentiating between subjects, it should be consistent within an individual, different between individuals and those differences should be measurable [18]. This requires knowledge of differences in gait features within and between subjects, i.e. of intra- and inter-subject variabilities.

### Intra- and inter-subject variabilities of gait

**Figure 1.**
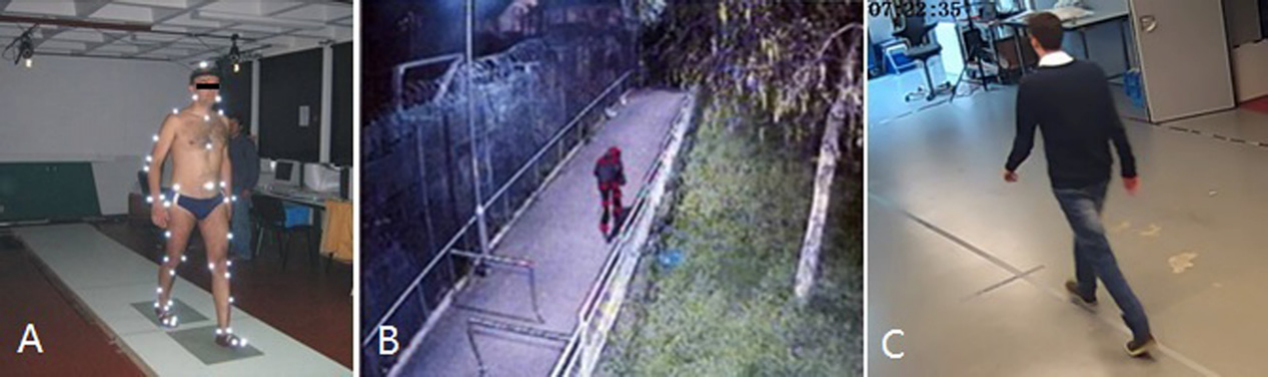
Three-dimensional motion analysis in a laboratory. (A) Clinical gait analysis [74]. (B) Manually labelled joint locations in surveillance video [3]. (C) Observer-based gait analysis [72] (with permission).

Whereas circumstances are uncontrolled in forensic gait analysis, most gait research is performed under controlled circumstances. The gold standard for measuring gait is three-dimensional (3D) motion analysis in a laboratory (Figure 1) [3,72,74]. Spatiotemporal characteristics such as step length and frequency, and kinematic variables such as joint and segment angles during the gait cycle can be calculated from marker positions on anatomical landmarks. Measurement accuracy highly depends on correct marker placement [19,20]: without correction, between-day variability (two marker placement sessions) is often higher compared with within-day variability (one session) [21,22]. The accuracy of quantifying most joint angles in 3D gait analysis is ±5° (standard deviation) [20].

3D motion analysis allows estimation of intra- and inter-subject variabilities in gait under constant circumstances. Since humans can adjust their gait to different circumstances, variability can be larger in practice. Factors affecting gait are summarized in Table 1.

**Table 1.** Potential factors influencing gait and forensic gait analysis.

Potential factors	Gait	Analysis
Internal	Gender [52–54]; Age [49,52,53,55,56]; Walking speed [27–30,49,57,58]; Injuries / physical limitations / surgeries [55]; Physical and mental disorders [49,55,59]; Emotions [45,49,59,60]; Posture and changes in body weight [49,54–56]; Pregnancy [55,61]; Alcohol/drunkenness [49,55]; Medication [62]; Drugs [63]; Music [49]; Visual information [64]	Type of camera [11]; Viewing angle/camera location [11,12,38,49,55,65–68]; Distance between camera and subject [3,^49^,^57^,^66^]; Frame rate [3,6,^11^,^12^,^38^,^57^]; Resolution [3,11,12,38,66]; Lens distortion [11,66]; Type of storage equipment [11,38]; Player specifications [11]; Elapsed time [49,55,^57^,^68^,^69^]; Number of observants [70]; Skills/experience of observants [12,38,71]; Interpretation differences [71,72]
External	Walking surface [49,55,65,68,69]; Type of clothing and/or footwear [3,12,49,55,57,65,68,72,73]; Carrying/propelling an object [49,53,55,68,69,73]; Elapsed time [49,55,57,68,69]	Illumination [3,11,12,38,49,55,57,65,67]; Shadow [3,12,^49^,^57^]; Reflections [12,57]; Indoor/outdoor environment [55]; Occlusion by individuals/objects [3,11,49]; Background and contrast [3,12,^49^,^57^]; Camera blur due to movement [49]; Type of clothing/footwear [3,12,^49^,^55^,^57^,^65^,^68^,^72^,^73^]

#### Intra-subject gait variability

Within a subject, walking at a fixed speed yields similar [21–23] and excellent (>0.90) [21,22] within- and corrected between-day reproducibility. The magnitude of this intra-subject variability has been found to be 1°–3° in healthy children for lower body angles [24]. Based on data of one adult subject, lower (1°–2°) within- [23] and between-day [23,25] variabilities were found. Wilken et al. [26] found higher between-day variabilities in 29 healthy adults of 1°–5°, but those values are not corrected for marker placement variation. Variability is mostly reflected in top-view angles, and to a lesser extent in front-view angles [21,22].

While intra-subject gait variability data are based on subjects walking at a constant speed, in daily life, humans use a range of walking speeds. Gait speed appears to be an important factor influencing joint angles. An increase in gait speed is accompanied by significantly increased flexion [27,28] but decreased extension [28] in the hip and knee. In the knee, stance phase [29] flexion and external rotation significantly increase, as well as ankle plantar flexion [30]. Minimum pelvic rotation and obliquity significantly increase [28]. However, sample sizes of the studies are limited and studies differ in speed conditions, the significance of speed effects, the derived variables and the magnitude of joint angle changes.

#### Inter-subject gait variability

Joint angles of subjects walking at a similar gait speed vary with a standard deviation of 2°–8° [26,27,30], although larger inter-subject variability was found for the minimum and maximum hip angle (around 13°) [28] and smaller inter-subject variability for pelvic rotation and obliquity (around 1.5°) [28].

In forensic practice, however, gait speed of a perpetrator and suspect are likely to differ. Therefore, Yang et al. [31] investigated whether and when in the gait cycle joint angles of a perpetrator and suspect can be compared if their speed differs. Importantly, in our opinion, comparing joint angles from two-dimensional (2D) video footage is meaningless with current techniques (see Forensic gait analysis methods). Nevertheless, we think that even for observer-based forensic gait analysis, knowledge of gait speed effects may be important. Yang et al. [31] found that joint angles were most invariant for gait speed at mid-stance and mid-swing (around 30% and 80% of the gait cycle). During the remainder of the gait cycle, especially at toe-off (50%–60% of the gait cycle), gait is too variable to compare joint angles separately. Front-view joint angles and higher gait speeds are more suitable for comparison than side-view angles and lower gait speeds [31]. Yang et al. [31] advise to compare gait joint angles of similar speeds if possible, and to select mid-stance or mid-swing video frames for comparison of joint angles otherwise.

Furthermore, in forensic practice, knowledge of the discriminative strength of gait features is important for discriminating individuals based on their gait. In children, Sangeux et al. [24] marked pelvic tilt, hip flexion and rotation angles as subject-specific joint angles based on an intra/inter-subject variability ratio of <40%. In adults walking multiple 3D gait analysis trials on two separate days, the rate of correctly matched trials of the same subject was 33%–90% based on lower-body kinematic time series [32]. Front-view variables had higher discriminative value compared to side- and top-view variables: a recognition rate of 100% was achieved combining three front-view angles of the thigh, shank and hip.

The latter result is in contrast with the study of Birch et al. [12], where forensic gait analysts achieved highest correct matching rates based on side-view recordings. The analysts attributed this to their reliance on arm swing for decision-making. The absence of upper-body kinematics in the study of Larsen et al. [32] and the different approach might explain this difference.

In conclusion, a requirement for using gait features in a forensics investigation is that features should be consistent within an individual and different between individuals. However, the (subjective) question when gait features are acceptably different between and consistent within subjects remains unanswered in literature. Some research exists on inter- and intra-subject variabilities: joint angles are reported to vary 1°–3° within a subject and 2°–8° between subjects. However, these numbers are based on small sample sizes. Research into the discriminative strength of different gait features is limited and not conclusive. Therefore, research should focus on expanding knowledge on inter- and intra-variabilities of gait features and their discriminative strength. This should be taken into account in forensic gait analyses when reporting evidential value of the analysis.

Since in forensic practice, circumstances vary (with gait speed being an important example), variability may be even larger. We, therefore, recommend comparing video clips of gait performed under similar circumstances if available or possible to collect covertly.

### Forensic gait analysis methods

Whereas in most gait research, 3D data are collected, forensic gait analysis concerns the extraction of gait features from 2D video footage captured under uncontrolled circumstances. To compare gait features with those measured in 3D motion analysis, a method could be to estimate joint angles from video footage by manually labelling joint positions as described in an observer-based forensic gait analysis [11]. Manual labelling of joint positions was also reported in a partly automated gait recognition approach to compare distance scores [3]. However, although the accuracy of quantifying joint angles from video footage has not been determined, it is at least less than the accuracy of 3D motion analysis (±5°) [18]. Probably, it is far less: accuracy of labelling joint positions is expected to be decreased by lower camera resolution (pixels), smaller subject size (meters) and a larger distance between subject and camera, as well as methodological prescriptions (Table 1). Furthermore, viewing angle influences the visibility of gait features, and the loss of a dimension (3D–2D) and resulting distortions will lower accuracy. Given inter-subject variability of 2°–8° [26,27,30], an intra-subject variability of 1°–3° [23–25], and an accuracy of >5°, the quantitative comparison of gait joint angles at a certain moment in the gait cycle cannot be considered meaningful with current techniques [18].

Two other types of forensic gait analysis have been proposed in the literature: automated gait recognition algorithms that require limited or no user intervention and observer-based forensic gait analysis (Figure 1).

#### Gait recognition algorithms

Current algorithms are either model-based [7] or appearance-based [8,9]. In the former approach, gait features like step length and (joint) angles are extracted by fitting a predefined human body model to each video footage frame of a walking individual [7]. In the latter approach, however, no prior knowledge about the human body is needed. Most model-free approaches derive silhouette sequences of the walking individual for use as a gait feature [7]. Based on these gait features, the algorithm calculates a match score between two video clips.

The algorithms are trained using video clips of walking subjects under controlled circumstances. Their performance is expressed as the classification accuracy or recognition rate [7]: the percentage correctly matched video clips. For both model- and silhouette-based algorithms high recognition rates in large datasets are reported: 80%–95% [7] and 94%–99% [9], respectively. While this seems promising, these algorithms have not been used in forensic casework yet, since variation inevitably occurring in real closed-circuit television (CCTV) footage (Table 1) results in a decrease of recognition rates [9].

To our knowledge, no fully automated gait recognition methods have been used in criminal cases yet. However, a partly automated gait recognition algorithm using manually selected joint positions for calculating a distance score has been used in practice [7]. A silhouette-based partly-automated gait recognition application has been developed for use in practice [8], with manual feature masking and interactive circumstance-dependent probability calculation. However, its use in casework has not been reported yet.

In our opinion, (partly-)automated methods are promising methods for video clips of similar circumstances. However, since this is often not the case, human analysts are still needed. In addition, we are cautious about model-based gait recognition algorithms relying on joint position estimation based on our concerns regarding accuracy and validity.

#### Observer-based forensic gait analysis

While many people would agree with the statement that humans are able to recognize individuals by their gait, the recognition rates of friends guessing each other’s identity based on side-view movies of joint positions of their gait [33] are only 36%–38% [34,35]. This is above chance level (16.7%) [34,35] but still low. Recognizing and discriminating strangers is not even above chance level [36]. Other views and methods might improve recognition and discrimination rates.

In a forensic context, gait observation is not used for direct recognition, but for comparison of a perpetrator and suspect(s). Observer-based forensic gait analysis encompasses systematic evaluation of the presence or absence of certain gait features (Table 2) from video footage. It originates from clinical research, with reliability depending on method, patient group and observer experience [37].

**Table 2.** Gait feature checklists.

General description	The Netherlands [67]	The United Kingdom [2–5]	Denmark [6]
Approach	3-point scale, only for visible features	3- to 10-choice scale, only for visible features	Notes; agreement/no agreement/incomparable
Foot and ankle^a^	Stance foot orientation: Outward/inward	Stance foot orientation: Outward/inward In/eversion Swing: Forefoot raise prior to heel strike Timing of heel raise Ankle abduction prior to heel strike	Foot outward rotation Ankle: Inversion/eversion Dorsal/plantar flexion at heel strike Degree of push-off
Knee^a^	Varus/valgus Extension/hyperextension at heel strike	Orientation and movement during swing: Inward/outward Flexion/extension/hyperextension: At heel strike Prior to heel rise	Varus/valgus Stance flexion
Hip^a^	Hip endorotation/exorotation	Hip movement: linear motion circumduction Thigh inversion in early stance phase	–
Pelvis	–	–	Ab/adduction, rotation, tilt
Upper body	Trunk sway: Amplitude Asymmetry	Head and torso motion: Frontal plane: Rolling Transverse plane: Yawing	Upper body: Lateral flexion of spinal column Forward/backward leaning Rotation
Shoulder	Horizontality	Relative height	Angle in frontal plane Forward/backward rotation
Head and neck	–	Head alignment relative to torso: Sagittal plane Frontal plane	Neck/head posture in sagittal plane Head movements in frontal plane
Other features	Step length differenc (L–R) Asymmetric body lift	Head and torso vertical movement per step^a^ Arm swing^a^ Symmetrical gait Base of gait width	Step length: Long/short^a^ Width between feet Smoothness of gait: Stiff/relaxed Signs of pathologic gait

L: left; R: right; –: no data.

^a^Both sides.

Birch et al. [12] showed that experienced forensic gait analysts matched a “target walker” correctly to one, multiple or none of five “suspect walker” video clips in 71% of the cases. Similar to gait recognition algorithms, correctly observing gait features is influenced by video clip characteristics (Table 1). Video clips with different viewing angles yield significantly lower correct match rates (*P* <0 .05) [12]. In addition, lower frame rates decrease feature observation performance [38]. To prevent inappropriate use of poor quality video footage, Birch et al. [39] developed a tool to assess suitability of footage for use in forensic gait analysis.

#### Gait databases

Uniqueness of gait is not essential for drawing forensic conclusions [40]: data on frequencies of gait features in the population can be used for calculating the likelihood of observing a specific combination of features. Therefore, gait feature databases are essential for estimating the likelihood ratio [41] of the hypotheses that the observed individuals have the same or another identity.

While gait databases are mentioned in the literature, they are currently not yet suitable for the calculation of likelihood ratios based on gait feature observations. Clinical gait databases differ significantly and disorders affecting gait will be either underrepresented or overrepresented [42]. Video databases designed for testing gait recognition algorithms contain video clips of subjects walking under controlled circumstances [43], but no gait feature frequencies. Therefore, these databases cannot be used in a forensic context.

Although observer-based gait feature databases have been used ad hoc in casework (see Part II) [13, 14], only one article was published on the collection of a forensic gait feature database [2]. One experienced gait analyst discreetly observed random pedestrians in seven public locations across the United Kingdom using a scoring sheet, resulting in a database consisting of 28 features of 1 007 British citizens. However, for correct calculation of likelihoods in casework, the relationship between gait features need further study [2]: if observed gait features are treated as completely independent features, the estimated prevalence in a population is lower than if features are known to have some level of dependency [2].

In conclusion, methods for analysing gait in forensic practice are either partly automated or observer-based. Although automated algorithms are promising, they still need human input and highly similar video clips. Attention must be paid to accuracy and validity of quantifying gait features from video footage. Observer-based methods are commonly used. However, for correct likelihood ratio estimation, the dependency between gait features must be investigated after designing and jointly collecting a set of most discriminative gait features.

### Conclusion

We share the concerns of Edmond and Cunliffe [16,17] on the limited scientific basis of forensic gait analysis at this moment. Fundamental knowledge of intra- and inter-subject variabilities, discriminative strength and interdependency of gait features is limited. Research into the use of this knowledge in forensic practice is developing but still limited. For observer-based methods, the ability of observers to score gait features should be investigated, as well as the persistence of these features in individuals. Gait feature databases should be expanded and (in)dependency of gait features should be determined for correct likelihood ratio calculation. In parallel, for automated methods, more attention should be paid to the accuracy and validity of quantifying gait features from video footage, as well as to handling with video clips of varying circumstances.

## Part II: The use of forensic gait analysis in practice

To evaluate the use of gait analysis, a survey was designed concerning the working process and challenges of forensic gait analysis. Participants were authors of scientific articles reporting the use of forensic gait analysis in casework or were investigators in current casework. Four gait analysts participated: one from the Netherlands (NL), one from Denmark (DK) and two from the United Kingdom (UK). Prof. Otten, the only registered candidate expert witness for gait analysis in the Netherlands, deals with about 10 cases a year. Dr Larsen (DK) is part of the only Danish research group for forensic gait analysis at the University of Copenhagen, who assist in about five criminal cases a year [6,10,11,31,32,44]. Two forensic gait analysis professors from the United Kingdom participated in the survey: Prof. Birch [1,2,12,38,39,45], who deals with about 30 cases a year in his forensic enterprise, and Prof. Nixon [3,4,46–49], who deals with maximally five cases a year using a partly automated gait recognition algorithm.

The results of the questionnaire cannot easily be generalized, since the amount of participants is low. On the other hand, based on our knowledge, we estimate the total amount of gait analysts in the three countries to be maximally 30.

### Investigation process

In most cases, gait analysts receive an investigation question from the police whether the gait features observed in questioned (perpetrator) and reference (suspect) footage could have come from the same or different individuals. The steps to answer this question are summarized in Table 3 for observer-based gait analysis.

**Table 3.** Process of observer-based forensic gait analysis.

Description	The Netherlands	The United Kingdom	Denmark
Orientation phase: video footage quality assessment: frame rate, viewpoint, walking direction, resolution	Exp0 (police) and/or exp1 + 2	If possible by exp0	Exp0 (police) and exp1, if needed also by exp2
Preparation phase: police instruction for reference footage	If needed (prior to interrogation)	If needed (in custody)	If needed (when brought in for interrogation)
Research phase: analysis and comparison of questioned (QF) and reference footage (RF)	QF by exp1 and exp2 independently	QF by exp1 and exp2 independently	QF and RF by exp1
	RF by exp1 and exp2 independently	RF by exp1 and exp2 independently	Discussing/modifying the assessment made by exp1 with exp2
	Comparison based on scores (and notes)	Comparison based on notes	Comparison based on notes
	Pooling 2 experts results	–	–
	Result program (Matlab)	–	–
Reporting and evaluation phase: reporting results and optionally further research	Writing report	Writing report	Writing report
	–	Independent verification of report by exp2	Present + discuss case exp1 with exp2
	Revision of report, if needed	Revision of report, if needed	Revision report, if needed
	Submission report (prosecutor/barrister)	Submission report (police/defence)	Submission report (police)
	Attend court (on request of prosecutor or defence)	Attend court (on request of prosecutor or defence)	Attend court (on request of prosecutor)
	Follow-up research, if needed	Additional research, if needed	–

Exp: expert. Exps 1 and 2 are gait analysts. Exp0 is an image analyst not involved in the gait analysis; –: no data.

In observer-based gait analysis, a scoring list is used to observe gait on the questioned footage (perpetrator) using a checklist of gait features (Table 3). All checklists include ankle, knee and hip features and orientation and motion of the trunk, shoulders and head, as well as symmetry and step length and width (Table 2). Features are scored on a multiple-choice scale and only visible features are used in the analysis.

Bouchrika et al. [3] use a partly automated gait recognition algorithm in which 10 joint positions are manually selected in each frame of both video clips. An average distance measure is calculated between joint positions in the fragments over joints and frames. This is compared with a threshold value to establish a confidence estimation.

All gait analysts stress that the answer to the research question is not an individualization, but an indication: the more gait characteristics shared by the individuals, the more likely it is that they have the same identity.

Both in the UK and DK, gait analysts provide a statement on the weight of the evidence on a standard verbal scale, along with their confidence in that statement. The statement is based on the expertise and experience of the gait analyst. In NL, however, the (combination of) gait feature frequencies observed in the population is estimated. An algorithm calculates the likelihood ratios of the hypotheses that the walking subjects on the video clips have the same or another identity using gait feature data of a random sample of >100 subjects. The verbal likelihood is derived from likelihood description standards [50].

### Challenges in the use of forensic gait analysis

Gait analysts mentioned practical challenges like problems with the playability of footage, low frame rates and partially visible individuals due to occlusion. They also mention the lack of a solid scientific knowledge base about intra- and inter-subject variabilities in gait features, as well as the influence of different internal and external factors on gait. They consider a gait database of features and their (combined) frequencies in the population essential for improving likelihood ratio estimations.

### Conclusion

We compared the criminal cases and survey results to the concerns of Edmond and Cunliffe [16,17].

First, forensic gait analysts indeed work in highly suggestive work conditions: analysts receive few video clips of suspect and perpetrator, containing more (i.e. domain-irrelevant) information than gait information alone [16,17], increasing the risk of cognitive and contextual bias. For example, confirmation bias refers to the human tendency to search for and interpret information confirming prior beliefs [51]. Anchoring effects refer to the tendency to rely on the first piece of information offered when making decisions [51]. Given these risks, it is important to assess the questioned footage prior to the reference footage by two observers independently, as is done in UK and NL. We support the recommendation of Edmond and Cunliffe [16,17] to design guidelines for minimizing the risks of cognitive bias.

Second, differences in evidence presentation were observed between gait analysts and courts. In DK and the UK, gait evidence is presented as expert opinion with confidence statement, whereas in the NL, likelihood ratios are calculated. However, these are not always admitted as evidence [14]. Analysts seem to have a large influence on the estimation of evidential value by the judge by stressing or not stressing the limitations of their method [15], showing video footage [10]. To improve clarity on admission of gait as evidence and assessing its evidential value, method validity and reliability and expert proficiency should be reported, as recommended by Cunliffe and Edmond [17]. In addition, guidelines should be designed to assist courts in this process [5].

It should be noted that although the risks for cognitive bias and the difficulty of assessing expert evidence are relevant to forensic gait analysis, these are general problems in forensic practice.

Contrary to the concerns of Edmond and Cunliffe [16], we found a high awareness of the limitations of forensic gait analysis among the participants in our questionnaire. They all stress its relatively low evidential value. Of course, expert behaviour and analysis quality differ, but again, this is not specific to forensic gait analysis. In addition, the concerns of Cunliffe and Edmond seem to be based on only two criminal cases [17] and only a selection of scientific literature [16], thus cannot just be generalized.

We hope that forensic gait analysts will join forces to create an international standard forensic gait analysis method with known validity, reliability and proficiency tests. We propose to focus on designing and publishing on large (inter)national gait databases and methods for likelihood calculation taking into account interdependent features. We also hope for (inter)national guidelines for the admission of forensic gait analysis in court. This is especially important since forensic gait analyst is not a protected professional title.

## Recent developments

Currently, a lot of developments are in progress within the field of forensic gait analysis, because of the existing challenges and limitations to use it as evidence in court.

**Figure 2.**
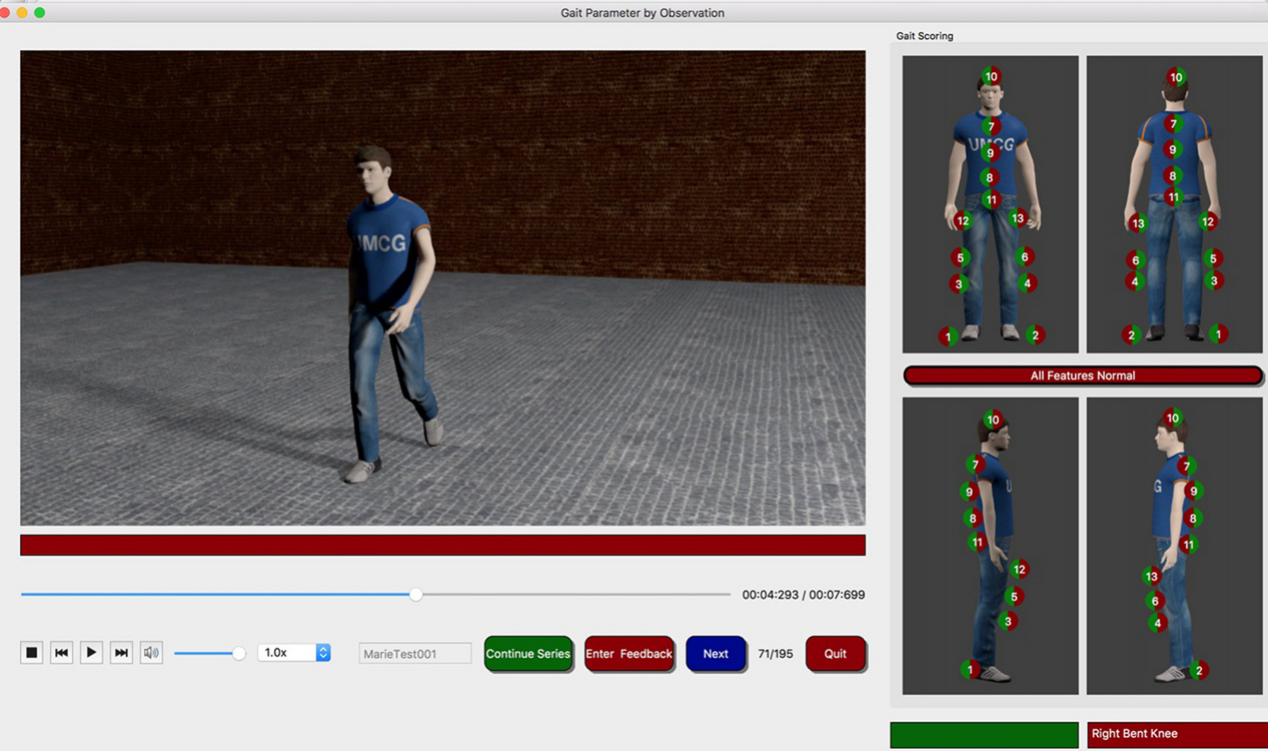
User interface of the Gait Observer Measurement Instrument (developers: Prof. E. Otten and M. Wiedemeijer, University of Groningen; 2017 e-mail from M. Wiedemeijer to authors; with permission).

In the field of observer-based gait analysis, in the UK, the group of Prof. Birch is currently evaluating the validity, repeatability and reproducibility of their gait feature scoring tool (2017 e-mail from I. Birch to authors; unreferenced). In the NL, the group of Prof. Otten is investigating the reliability and trainability of observers and the detectability of specific gait features from different camera viewpoints using avatar animations of gait in the Gait Observer Measurement Instrument (GOMI) (2017 e-mail from M. Wiedemeijer to authors; unreferenced) (Figure 2).

In the UK, Prof. Nixon and his group are focusing on metric-based automated gait analysis methods and the evaluation of biometric-based evidence for admission in court [7]. The group collaborates with the Australian police (2018 e-mail from M. Nixon to authors; unreferenced).

In the NL, the Netherlands Forensic Institute (NFI) and Prof. Otten collaborate to publish a solid statistical calculation of likelihood ratios based on gait feature observations. Prof. Birch is working on a project to use likelihood ratios for forensic gait analysis in the UK in future (2018 e-mail from I. Birch to authors; unreferenced).

The need for guidelines for gait analysis has been recognized in the United Kingdom. Recently, a “primer for courts” was written by scientists and judiciary members to assist judges when handling evidence from gait analysis in the courtroom [18]. It presents an easily understood summary of forensic gait analysis, explaining its limitations and challenges in application. However, particularly its comments on likelihood ratios have resulted in some discussion. In addition, the Forensic Gait Analysis Working Group of The Chartered Society of Forensic Sciences is writing a standard for forensic gait analysis, for the Forensic Science Regulator of the United Kingdom (2017 e-mail from S. Reidy to authors; unreferenced) as the initiating party. These standards will be published in the near future.

## Conclusion

In this review, we summarized the scientific basis of forensic gait analysis and evaluated its use in the NL, UK and DK, following critique of Edmond and Cunliffe [16,17] on the admission of gait evidence. Gait features for differentiating between individuals should be (1) measurable, (2) consistent within and (3) different between individuals [18]. Reviewing scientific literature, we found that (1) gait features can be quantified or observed from surveillance video footage by (partly-) automated gait recognition algorithms and observers. Whereas algorithms seem promising, their suitability for use in practice is currently limited. Observer-based methods are currently used, but gait feature databases and likelihood estimations should be improved. Information on accuracy, validity and reliability of the methods is limited. Gait is variable (2) within individuals under differing and constant circumstances, with speed having major influence. However, joint angle variability data are based on small sample sizes. Although clearly variation exists between individuals, research on the discriminative strength of gait features (3) is limited and not conclusive. Therefore, we agree with Edmond and Cunliffe [16,17] that the scientific basis of forensic gait analysis is currently limited. However, it should be noted that the amount of scientific literature on this topic is larger than suggested by Edmond and Cunliffe [16].

Nevertheless, forensic gait analysis has been used as supportive evidence in several criminal trials in Europe in the past 15 years, mostly based on the congruence between observed gait features of perpetrator and suspect(s). Evidence presentation (verbal or likelihood), analysis quality and expert quality can differ between criminal cases. Although the concerns of Cunliffe and Edmond [17] are based on only two criminal cases, we also think that the admission of gait as evidence should be clarified. We also share their concerns regarding the risk of cognitive and contextual bias. However, we do not fully agree with the concerns of Edmond and Cunliffe [16] on the awareness of limitations of forensic gait analysis among gait analysts, since in our survey participants all stress the relatively low evidential value of forensic gait analysis. Of course, other gait analysts could be less aware of the limitations.

The use of forensic gait analysis could be improved by

- Scientific studies to expand knowledge on intra- and inter-subject gait feature variabilities and discriminative value and interdependency of measured or observed gait features, and clarify the collection and use of databases and likelihood estimation calculations.
- Scientific studies to compare strengths and limitations of model- and silhouette-based (partly-) automated gait recognition algorithms with observer-based methods, and evaluate whether and when they should be used complementary or individually.
- An international standard method with known accuracy, validity and reliability and proficiency tests for gait analysts.
- An international standard data collection method for gait feature databases, resulting in analogous (inter)national gait feature databases.
- (Inter)national guidelines for the admission of gait evidence in court.
- Special attention to decreasing the risk for cognitive and contextual bias in forensic gait analysis.

This is expected to improve admission of gait evidence in court and assessment of its evidential value. We think these recommendations and current research projects will contribute to more theoretically substantiated gait analysis methods and its appropriate use in future criminal cases.
